# Radiographic findings in acromegaly: pictorial essay

**DOI:** 10.1590/0100-3984.2022.0083-en

**Published:** 2023

**Authors:** Luisa Couto Baptista, Marcelo Mantiolhe Martins, Vinícius Neves Marcos

**Affiliations:** 1 Department of Radiology, Hospital Universitário da Universidade Federal de Juiz de Fora/Empresa Brasileira de Serviços Hospitalares (HU-UFJF/EBSERH), Juiz de Fora, MG, Brazil

**Keywords:** Acromegaly, Arthralgia, Radiography, Acromegalia, Artralgia, Radiografia

## Abstract

Acromegaly is an uncommon metabolic disorder, often diagnosed after a long delay. One symptom seen in many patients with acromegaly is arthralgia, a finding that calls for the use of conventional radiography, which can reveal subtle changes that can go unnoticed. The objective of this pictorial essay is to portray the radiographic aspects of acromegaly, seeking to demonstrate the importance of conventional radiography, which, despite its simplicity, can suggest the diagnosis, even in the early stages, thus altering the clinical course of the disease.

## Abstract (pt)

A acromegalia é um distúrbio metabólico pouco frequente, muitas vezes apresentando grande atraso diagnóstico. A artralgia é um dos sintomas mais comumente encontrados nos pacientes acromegálicos, motivando a realização de radiografias convencionais, as quais podem apresentar achados sutis que podem passar despercebidos. O objetivo deste ensaio é retratar os achados radiográficos da acromegalia, buscando demonstrar a importância deste método que, apesar de simples, pode sugerir o diagnóstico, mesmo em fases iniciais, alterando assim o curso clínico da doença.

## INTRODUCTION

Acromegaly is a rare metabolic disorder with an insidious evolution, distinguished by chronically elevated circulating levels of growth hormone and insulin-like growth factor 1, which can lead to high morbidity and mortality^(1,2)^. It is estimated that there is an average delay of seven to ten years from the onset of symptoms to the diagnosis of the disease^(1,3)^, which contributes to the onset or exacerbation of comorbidities, as well as worsening quality of life and increasing mortality, mainly due to an increase in cardiovascular risk. In addition, due to the bone and skin deformities it causes, acromegaly is associated with psychosocial impairment, including depression, body dysmorphic disorder, and social isolation^(1,4)^.

Arthralgia is a prevalent complaint in patients with acromegaly, usually resulting from arthropathy, a condition seen in up to 70% of cases. Typically, arthralgia prompts a request for conventional radiography, which, because of its low cost and wide availability, is often the first examination to be performed in such patients^(5,6)^.

Although acromegaly has multiple radiographic features, they may go unnoticed when the diagnostic suspicion not been raised or when the changes are subtle and incipient. Therefore, the recognition of radiographic changes is extremely important for the early diagnosis and treatment of acromegaly, improving the prognosis as well as avoiding sequelae such as deformities and stigmata^(6,7)^.

The aim of this pictorial essay is to present the various aspects of acromegaly seen on conventional radiography and to demonstrate the importance of such method, which, albeit simple, is capable of suggesting the diagnosis of acromegaly and thus altering the clinical course of the patients.

## RADIOGRAPHIC FINDINGS IN ACROMEGALY

The effects of growth hormone on the skeleton vary depending on the degree of skeletal maturation. When the growth plates are still open, hormonal excess will promote direct stimulation of the endochondral bone and consequent proportionate bone growth, in length and in width (gigantism). In the mature skeleton, in which the growth plates are closed, hypersecretion of growth hormone can result in reactivation of endochondral bone formation at the junction sites between cartilage and bone, such as the costochondral junctions, as well as promoting periosteal reactions, with consequent bone enlargement^(8)^. Conventional radiography, despite being the least complex imaging method, is of great importance for raising the diagnostic suspicion of acromegaly, the clinical characteristics of which typically have corresponding radiographic alterations that are quite suggestive of the diagnosis.

### Radiographic alterations in joints

**Figure 1:**
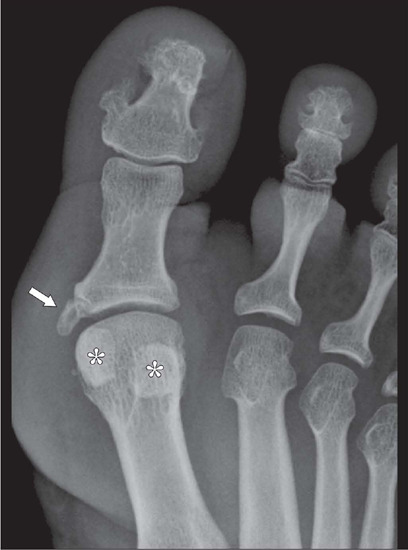
Radiograph of the left hip, in an anteroposterior view, showing an increase in the femoroacetabular joint space in a patient with acromegaly (**A**), compared with the normal space in a healthy patient (**B**).

In patients with acromegaly, arthropathy can be caused by two different mechanisms: hormonal and mechanical. Initially, the high concentrations of growth hormone and insulin-like growth factor 1 would promote hypertrophy and anabolism of the joint connective tissue, as well as of the periarticular ligaments. Clinically, the joint may present limited mobility, due to cartilage thickening, or even laxity, due to the excessive growth of ligaments, translating to widening of the joint spaces on radiographs (Figure 1). At that stage, it is presumed that the changes can be reversed through pharmacological and surgical treatment of the underlying disease^(5)^.

**Figure 2:**
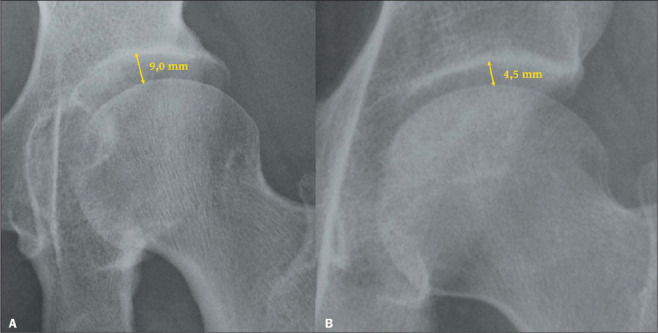
Radiograph of the hallux of a patient with acromegaly, showing a coarse, beak-like enthesophyte (arrow) at the base of the proximal phalanx, the insertion site of the medial collateral ligament of the metatarsophalangeal joint. Note also the hypertrophy of the sesamoid bones (asterisks).

Over the course of acromegaly, especially uncontrolled acromegaly, the morphological and architectural distortion of the joint results in repeated intra-articular trauma and consequent exaggerated repair responses, which translate, on radiographs, to narrowing of the joint space, osteophytosis, the formation of subchondral cysts, and sclerosis. At that stage, the alterations become irreversible, in a manner very similar to that observed in osteoarthrosis^(5)^. The joints most commonly affected in acromegaly are the glenohumeral, femorotibial, and femoroacetabular joints, followed by the elbow, hand, and foot joints^(8)^. In addition, bony excrescences and enthesophytes (bone spurs), caused by stimulation of bone formation, can be seen at tendon or ligament attachment sites^(8)^, as can beak-like enthesophytes (Figure 2), which can arise in various locations, even in the pubic symphysis^(8)^.

### Radiographic alterations in the skull

**Figure 3:**
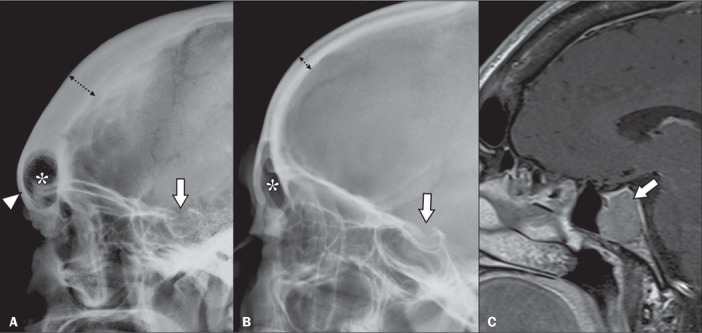
**A:** Radiograph of the skull, in a lateral view, of a patient with acromegaly, showing increased thickness of the frontal bone (dotted arrow) and forehead prominence (arrowhead), as well as increased dimensions of the frontal sinus (asterisk) and enlargement of the sella turcica (arrow). **B:** Lateral radiograph of the skull of a healthy patient, with preserved frontal bone thickness (dotted arrow), as well as normal dimensions of the frontal sinus (asterisk) and sella turcica (arrow). **C:** Gadolinium contrast-enhanced, fat-saturated, sagittal T1-weighted magnetic resonance imaging scan of the same patient depicted in panel **A**, showing pituitary macroadenoma (arrow), which was responsible for the enlargement of the sella turcica.

As illustrated in Figure 3, the radiographic manifestations of acromegaly in the skull include a series of alterations^(8,9,10)^. Some, such as enlargement of the sella turcica, are due to the direct action of the pituitary tumor. Others are due to the effect of excessive periosteal bone formation and include prominence of the occipital protuberance; enlargement and elongation of the mandible; an increase in the mandibular angle; cortical thickening of the cranial vault; enlargement of the maxillary and frontal sinuses, and the formation of a supraorbital crest. Clinically, those changes result in marked prominence of the forehead.

### Radiographic alterations in the hands, wrists, and feet

**Figure 4:**
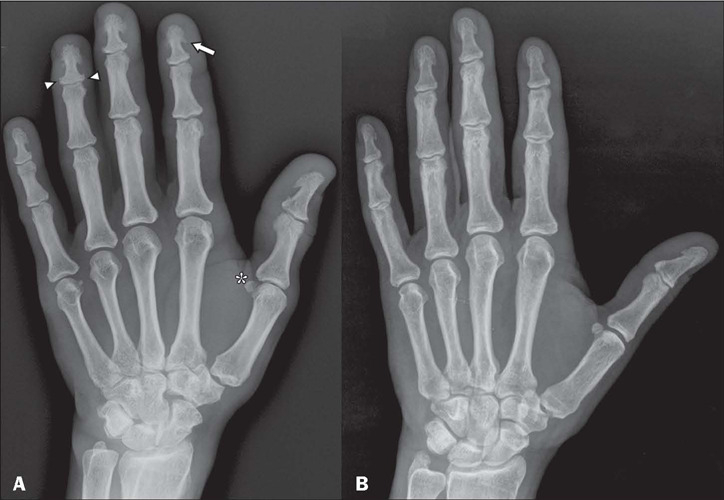
**A:** Conventional radiograph, in an anteroposterior view, of the right hand of a patient with acromegaly, showing hypertrophy of the terminal tufts of the distal phalanges, forming projections similar to spurs (arrow), as well as enlargement of the bases of those phalanges (arrowheads) and a slight increase in the size of the sesamoid bone (asterisk). Note also the prominence of the soft tissues around the fingers. **B:** Conventional radiograph, in an anteroposterior view, of the right hand of a healthy patient, for comparison.

**Figure 5:**
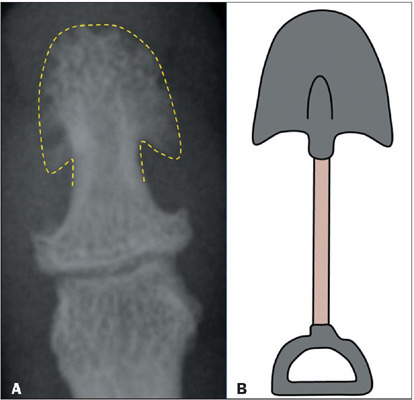
**A:** Radiograph showing the spade-like appearance of the distal phalanx, caused by enlargement and hypertrophy of the terminal tuft, in the hand of a patient with acromegaly. **B:** Schematic drawing of a spade.

**Figure 6:**
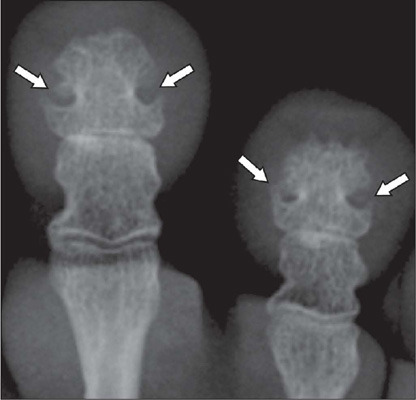
Radiograph of the distal phalanges in the foot of a patient with acromegaly, showing marked hypertrophy of the bases and terminal tufts, leading to the formation of a pseudoforamen (arrowheads).

**Figure 7:**
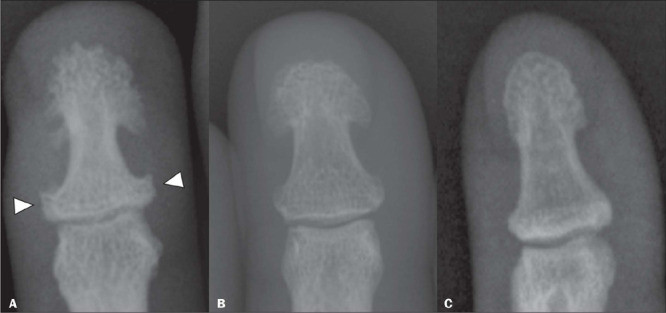
**A:** Radiograph of the distal phalanx of the hand of a patient with acromegaly, showing hypertrophy of the terminal tuft, with a spade-like appearance, together with widening of the base of the phalanx (arrowheads). **B:** Radiograph of the distal phalanx with a spade-like appearance but without significant widening of the base, suggestive of a normal variant. **C:** Radiograph of the distal phalanx in a healthy patient, showing no alterations.

**Figure 8:**
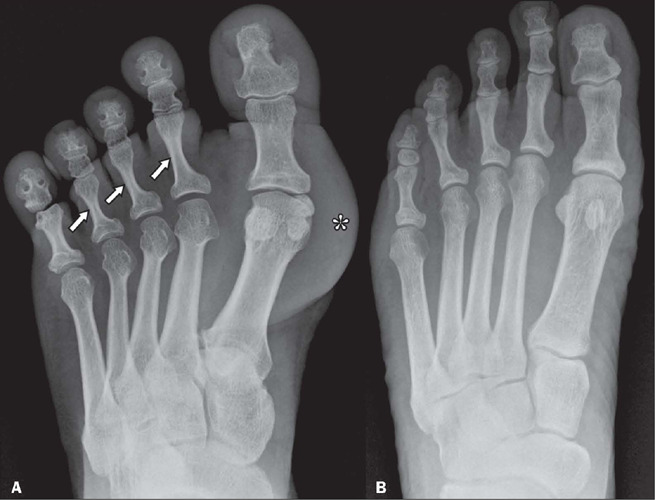
Radiographs illustrating abnormalities of the foot. Note the hyperconstriction of the proximal phalanges of the second to fourth fingers of a patient with acromegaly (arrows in **A**) in relation to those of a healthy patient (**B**). Additional findings in the patient with acromegaly included soft tissue enlargement (asterisk), prominence of the tufts and bases of the terminal phalanges, a pseudoforamen, enlargement of some of the metatarsophalangeal joints and prominence of the sesamoid bones of the hallux.

Characteristically, acromegaly causes thickening of the soft parts of the fingers due to a direct hormonal effect, together with enlargement of the sesamoid bones, phalanges, and metacarpals, mainly of the terminal tufts of the distal phalanges, resulting from excessive periosteal bone formation^(8,10)^, as depicted in Figure 4. Hypertrophy of the terminal tufts of the distal phalanges results in a classic spade-like appearance (Figure 5). When accompanied by pronounced enlargement and hypertrophy of the phalangeal base, that can lead to the formation of a pseudoforamen (Figure 6). Although enlargement and hypertrophy of the terminal tufts of the distal phalanges are characteristic signs of acromegaly, they can be present as normal variants (Figure 7), being more common in men who perform manual labor and in the elderly^(8)^. As shown in Figure 8, there can also be hyperconstriction of the phalangeal diaphyses, caused by simultaneous formation and resorption of bone^(8)^.

### Radiographic alterations in soft tissues

**Figure 9:**
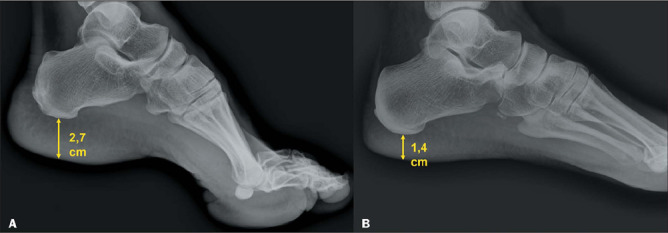
Radiograph of the foot, in a lateral view, showing increased thickness of the plantar fat pad of the foot of a patient with acromegaly (**A**) compared with that of a healthy patient (**B**).

Hormonal hypersecretion stimulates collagen synthesis in soft tissue, resulting in thickening of the skin (Figure 9), especially below the calcaneus^(8,10)^. Although the degree of thickening varies depending on the sex and ethnicity of the individual, skin thickness greater than 2.5 cm in men and 2.3 cm in women is considered highly suggestive of acromegaly^(8)^.

### Radiographic alterations in the spine and rib cage

Due to excess periosteal bone formation, patients with acromegaly can also show increases in the sagittal and transverse diameters of the vertebral bodies, as well as in their posterior concavity^(8,9)^. For the same reason, there can be prominent, diffuse osteophytic changes in the vertebrae that can even resemble those seen in ankylosing spondylitis and diffuse idiopathic skeletal hyperostosis. Other important findings resulting from the stimulation of endochondral bone formation and hormonal activity in connective tissue are intervertebral disc thickening, ligament laxity, spinal hypermobility, and thoracic hyperkyphosis^(8)^.

Stimulation of endochondral bone formation can cause the costochondral junctions to widen, giving the thorax a broader appearance. Elevation of the lower portion of the sternum and an increase in the sternal angle can also be observed^(8)^.

## CONCLUSION

Despite the fact that acromegaly is a disease with highly suggestive clinical characteristics, it is still not well known in the medical field, which results in significant delays in its diagnosis and, consequently, its treatment, thus worsening the prognosis and patient quality of life. The early diagnosis of acromegaly requires that health professionals be aware of the typical clinical manifestations, as well as the radiographic findings, which can be subtle in the early stages of the disease.
